# Admixture in Africanized honey bees (*Apis mellifera*) from Panamá to San Diego, California (U.S.A.)

**DOI:** 10.1002/ece3.8580

**Published:** 2022-02-14

**Authors:** Daniela Zárate, Thiago G. Lima, Jude D. Poole, Erin Calfee, Ronald S. Burton, Joshua R. Kohn

**Affiliations:** ^1^ Section of Ecology, Behavior, and Evolution Division of Biological Sciences University of California, San Diego La Jolla California USA; ^2^ Scripps Institute of Oceanography University of California, San Diego La Jolla California USA; ^3^ Division of Biological Sciences University of California, San Diego La Jolla California USA; ^4^ Department of Evolution and Ecology and Center for Population Biology University of California, Davis Davis California USA

**Keywords:** admixture, Africanized honey bees, *Apis mellifera*, genetic diversity, hybridization

## Abstract

The Africanized honey bee (AHB) is a New World amalgamation of several subspecies of the western honey bee (*Apis mellifera*), a diverse taxon historically grouped into four major biogeographic lineages: A (African), M (Western European), C (Eastern European), and O (Middle Eastern). In 1956, accidental release of experimentally bred “Africanized” hybrids from a research apiary in Sao Paulo, Brazil initiated a hybrid species expansion that now extends from northern Argentina to northern California (U.S.A.). Here, we assess nuclear admixture and mitochondrial ancestry in 60 bees from four countries (Panamá; Costa Rica, Mexico; U.S.A) across this expansive range to assess ancestry of AHB several decades following initial introduction and test the prediction that African ancestry decreases with increasing latitude. We find that AHB nuclear genomes from Central America and Mexico have predominately African genomes (76%–89%) with smaller contributions from Western and Eastern European lineages. Similarly, nearly all honey bees from Central America and Mexico possess mitochondrial ancestry from the African lineage with few individuals having European mitochondria. In contrast, AHB from San Diego (CA) shows markedly lower African ancestry (38%) with substantial genomic contributions from all four major honey bee lineages and mitochondrial ancestry from all four clades as well. Genetic diversity measures from all New World populations equal or exceed those of ancestral populations. Interestingly, the feral honey bee population of San Diego emerges as a reservoir of diverse admixture and high genetic diversity, making it a potentially rich source of genetic material for honey bee breeding.

## Graphical abstract

Here, we assess the nuclear genomic admixture, mitochondrial origins, and genetic diversity measures of Africanized honey bees (AHB) from four populations stretching from the isthmus of Panamá to San Diego, California. We find that AHB nuclear genomes from Central America and Mexico have predominately African genomes but honey bees from San Diego emerge as a reservoir of diverse admixture and high genetic diversity, making it a potentially rich source of genetic material for honey bee breeding.

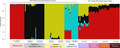

## 1 INTRODUCTION

Hybridization, the interbreeding of distinct genetic lineages, has long complicated taxonomic boundaries and challenged the perception of species as discrete taxonomic and evolutionary units. Initially considered an evolutionary dead end, hybridization is now recognized as a driver of adaptation and diversification across various evolutionary lineages. Sunflower (*Helianthus*) hybrids colonize habitats prohibitive to parental species (Rieseberg et al., 2003; Whitney et al., 2015). Admixture jumpstarted the spectacular diversification and adaptive radiation recognized in African cichlids (Seehausen, 2004). Interbreeding between extinct Denisovans and ancient *Homo sapiens* facilitated the transfer of high‐altitude adaptation genes found in contemporary Tibetan peoples—an adaptation paralleled in highland wolves and their domesticated dog counterparts (Huerta‐Sánchez et al., 2014; VonHoldt et al., 2017). Whether admixture generates creative evolutionary novelty (Barton, 2001; Hedrick, 2013; Suarez‐Gonzalez et al., 2018), or leads to destructive cellular incompatibilities (Burton & Barreto, 2015; Dobzhansky, 1935), hybridization dynamics are of great eco‐evolutionary interest, particularly in the context of increasing rates of unnatural, human‐mediated hybridization (HMH) (reviewed in Grabenstein & Taylor, 2018).

The Africanized honey bee (AHB) is a human‐mediated hybrid of the American continents, created from the intercross between an African subspecies (*Apis mellifera scutellata*) and various European and Middle Eastern honey bee subspecies. The western honey bee (*Apis mellifera*) is taxonomically diverse, comprising over thirty recognized subspecies traditionally clustered into four major, lineages based on genetic, geographic, and morphometric data: A (African), M (Western European), C (Eastern European), and O (Middle East and Anatolia) (Cridland et al., 2017; Ruttner, 1988; Wallberg et al., 2014; Whitfield et al., 2006). Recently, new lineage groupings have been proposed: Y, for bees from the Arabian Peninsula (see, e.g., Cridland, Tsutsui, et al., 2017) and Z (Alburaki et al., 2013) referring to bees of the traditional O clade from Syria. Here, we are particularly interested in which honey bee lineages contribute to New World bee populations and we use the A, M, C, and O nomenclature because known importations of bees to the New World come from these clades (Carpenter & Harpur, 2021; Ruttner, 1988). Substantial variation in behavior, morphology, and genetics exists across honey bee subspecies, even within the overarching clades. The Eastern European subspecies (C clade) are particularly favored in commercial beekeeping due to their gentle nature and predilection for honey production. In contrast, African subspecies are largely disfavored for both commercial and hobbyist use due to the intensity of their nest defense and high propensity to abscond (abandon the nest *en masse* and move to another (Ruttner, 1988)).

Early honey bee importations to the Americas were largely Western European (M) and Eastern European (C) in origin (reviewed in Schneider et al., 2004). Generally, African (A) subspecies were excluded from importation with modest exceptions (see Schiff & Sheppard, 1993). However, temperate‐adapted, non‐native European honey bees (EHB) struggled to thrive in the Neotropics. In response, honey bee researchers in Sao Paolo, Brazil initialized a breeding program in 1956, importing 47 queens of the African subspecies (*A. m. scutellata*) for experimental crossing (reviewed in Schneider et al., 2004). Researchers bred this African subspecies with European races, hoping to forge a superior hybrid for tropical beekeeping. These hybrid “Africanized” honey bees (AHB) escaped from their experimental apiaries and spread into the surrounding countryside (reviewed in Schneider et al., 2004).

The expansion of the AHB across the American continents over the past 60+ years is considered one of the “most spectacular biological invasions of all time” (Pinto et al., 2005). From their original Brazilian epicenter, AHB spread across South and Central America, rapidly hybridizing with and replacing the pre‐existing European honey bee population with one of predominantly African ancestry (reviewed in Schneider et al., 2004 and references therein; Whitfield et al., 2006; Nelson et al., 2017; Cridland, Tsutsui, et al., 2017). On the southern front, AHBs reached their range limit in Argentina in the 1970s at approximately 34° south latitude, presumably stopped from advancing further by the colder climate (Taylor & Spivak, 1984). In their northern expansion, AHB reached Panamá by 1982, Costa Rica by 1986, Mexico by 1989, Texas by 1990, and California by 1994 (Kim & Oguro, 1999). Currently, African mitochondria and nuclear markers are present in feral honey bees in California as far as 38° north latitude (Calfee et al., 2020; Kono & Kohn, 2015; Lin et al., 2017). While the current northern range limit may not be stable in the face of climate change, northward range expansion has clearly slowed in comparison to its explosive (160–500 km/year) neotropical expansion (Schneider et al., 2004 and references therein).

The dramatic shift from European to predominantly African ancestry throughout the majority of the New World suggests that Africanization provides ecological advantages in the areas where it dominates. Several behavioral and physiological traits of the Africanized honey bee are thought to drive AHB success: high reproductive rates; intense nest defense (McNally & Schneider, 1992a, 1992b, 1996; Fewell & Bertram, 2002; see also Breed et al., 2004); and higher resistance to infestation from the mite (*Varroa destructor*), a parasite and disease vector implicated in honey bee nest failure (Goulson et al., 2015; Guzman‐Novoa et al., 1996). The advantages, if any, AHB derives from its remaining European ancestry are less clear although Nelson et al. (2017) identified European genes underlying ovary size inferred to be selected for and Harpur et al. (2020) showed that both African and European ancestry underlies AHB nest defense.

In this study, we characterize the admixture and genetic diversity of AHB in the Neotropics and the southwestern United States using a dataset of 60 high depth (~25×) AHB whole genome sequences (WGS) collected from four regions spanning ~6000 km. Each sampling site reflects a distinct time since initial contact between resident European and advancing Africanized forms: the isthmus of Panamá; Guanacaste NP, Costa Rica; Chiapas, Mexico; and San Diego County, CA, U.S.A. We leverage two existing sequencing projects (Harpur et al., 2014; Wallberg et al., 2014) and the recently published honey bee reference genome with chromosome‐length scaffolds (Wallberg et al., 2019) for our analyses. This is the first genomic study to assess ancestry in AHB samples from Central America and Mexico as well as the first to assay the contribution of the O lineage in the regions sampled. The contribution of this lineage to the California honey bee population was not evaluated in previous genomic studies (Calfee et al., 2020; Cridland et al., 2017) and is of interest because mitochondria from this lineage are known to be present at considerable frequency in southern California's feral honey bees (Kono & Kohn, 2015) and occasionally elsewhere in the United States (Magnus & Szalanski, 2010). Ultimately, our study aims to broaden our understanding of the admixture dynamics of a massive hybrid takeover of an invasive social insect of great agricultural importance.

## 2 MATERIALS AND METHODS

### 2.1 Sample collection

We collected 60 Western honey bees (*n* = 15/country) from sites in each of four countries: the isthmus of Panamá; Guanacaste National Park, Costa Rica; Chiapas, Mexico; San Diego County, California, U.S.A. (Table 1). All samples were collected in June 2015–August 2016 by hand‐netting. Honey bees in Panamá were collected with an insect net while they foraged either on natural vegetation in rural areas, or on street vendor syrup dispensers in urban areas. Honey bees were collected across the isthmus of Panamá from five sites, each separated by >5 km: Panamá City, Gamboa, Barro Colorado Island (BCI), Santa Rita Arriba, and Cólon. Individuals from Costa Rica were collected from the Santa Rosa sector of Guanacaste National Park in northwestern Costa Rica. These bees were collected from a localized region and likely originate from a small number of feral colonies. Honey bees from Mexico were collected from an apiary in the southern state of Chiapas, with each bee collected from a different hive. Honey bees from San Diego County, California, U.S.A. were workers collected while foraging on flowers. San Diego bees were collected across 15 sites each separated by >5 km so that each likely represents a worker from a different colony. The furthest collection sites were separated by 65 km. Collection sites ranged from urban to rural settings. Due to the presence of hobbyist and agricultural beekeeping we do not rule out the possibility that the captured honey bees were from managed rather than feral hives. However, most honey bee foragers in San Diego are from feral hives (Kono & Kohn, 2015, and see results).

**TABLE 1:** Summary of all 159 genomes included in this ancestry analysis, including (A) 99 reference honey bee genomes downloaded from NCBI from Wallberg et al. (2014) and Harpur et al. (2014). (B) 60 admixed honey bee genomes collected from four distinct sampling sites

(A)				
Clade	Subspecies	(*n*)	Source country	Sequencing project
A	*A. m. scutellata*	21	South Africa	Wallberg et al. (2014), Harpur et al. (2014)
M	*A. m. mellifera*	15	England, Poland	
	*A. m. iberiensis*	14	Ireland, Spain	
C	*A. m. carnica*	19	Italy, Germany, Croatia, Slovenia	
	*A. m. ligustica*	10	Greece	Wallberg et al. (2014)
O	*A. m. syriaca*	10	Syria	
	*A. m. anatoliaca*	10	Lebanon	

(B)		
Location	(*n*)	Coordinates
San Diego, CA, U.S.A.	15	32.7157°N, 117.1611°W
Chiapas, Mexico	15	16.7569°N, 93.1292°W
Santa Rosa National Park, Costa Rica	15	10.8379°N, 85.7051°W
Panamá City, Panamá	15	9.1521°N, 79.8465°W

### 2.2 Reference honey bee genomes

Reference honey bee genomes were obtained by downloading 89 whole genomes sequenced by two previously published sequencing projects and made available on NCBI: Wallberg et al. (2014) (Project ID: PRJNA236426) and Harpur et al. (2014) (accession no. SRP029219). The reference genomes sequenced by Wallberg et al., 2014 were generated by whole genome sequencing on a SOLiD 5500xl platform to produce 75‐bp reads with an average coverage of 4.4× ± 1.5× per individual (Wallberg et al., 2014). The reference genomes sequenced by Harpur et al. (2014) were sequenced using Illumina Hi‐Seq to produce 50‐bp reads with an average coverage of 38×. Between both sequencing projects, we obtained 21 African (A) genomes of the subspecies *A. m. scutellata*; 29 genomes from the Western (M) clade, comprising two subspecies: *Apis mellifera mellifera* (*n* = 14) and *Apis mellifera iberiensis* (*n* = 15); 29 genomes of the Eastern European clade (C) represented by the subspecies: *Apis mellifera carnica* (*n* = 19) and *Apis mellifera ligustica* (*n* = 10) and 20 genomes from the Middle Eastern (O) clade, including the subspecies: *Apis mellifera anatoliaca* (*n* = 10) and *Apis mellifera syriaca* (*n* = 10) (see Tables 1, S1). In total, we used a panel of 89 reference honey bee genomes representing the four major honey bee clades and spanning 7 subspecies.

### 2.3 DNA extraction & sequencing

We extracted DNA from crushed heads of the 60 sampled honey bees using the standard protocol of the Qiagen DNAeasy Blood & Tissue extraction kit. DNA purity and appropriate concentration for sequencing were validated with a Qubit fluorometer prior to submission for library preparation. The DNA was submitted for DNA KAPA library construction and whole‐genome sequencing at the Institute for Genomic Medicine (IGM), UC San Diego. All 60 individuals were multiplexed and sequenced across three lanes of an Illumina HiSeq4000 platform to produce 100‐bp paired end reads. Average genomic coverage per individual was 29 ± 1.2×.

### 2.4 Sequence filtering & alignment

Raw reads generated from sequencing, and those downloaded from NCBI, were trimmed and filtered for quality and length using a PoPoolation (Kofler et al., 2011) perl script (trim‐fastq.pl) (settings: –fastq‐type sanger ‐‐quality‐threshold 25 ‐‐min‐length 40). Filtered reads were aligned to the most recently assembled honey bee reference genome Amel_HAv3.1 with chromosome‐length scaffolds (Wallberg et al., 2019) using the BWA v0.7.12 bwa mem algorithm under default settings (Li & Durbin, 2009). Reads were then sorted, merged, and filtered again for mapping quality (quality score <20 were discarded) using Samtools (Li, 2011). Read duplicates were removed using GATK Picard Tools' Remove Duplicates function (Picard Tools).

### 2.5 Variant calling and genotype likelihood estimation

We used the program ANGSD v0.930 (Korneliussen et al., 2014) to call variant sites and estimate genotype likelihoods (‐‐doGlf 2) across all 159 honey bee genomes. The major and minor alleles were inferred (‐‐doMajorMinor 1) as follows. A threshold likelihood ratio for SNP calling was set (‐‐SNP_pval 1e‐6) and allele frequencies were calculated using inferred major and minor alleles (‐‐doMaf 1). In addition, we discarded reads with a mapping quality below 30 and a base quality below 20. We removed tri‐allelic sites and only included sites in which we had at least 63% of individuals reporting information with a depth of coverage of at least 3×. We chose a genotype likelihood approach over a called genotype approach such as that used by other ancestry software progams like ADMIXTURE (Alexander et al., 2009) as genotype likelihoods have been shown to be robust to low‐coverage sequencing data (Korneliussen et al., 2014; Skotte et al., 2013). This SNP set was then thinned for linkage disequilibrium, keeping 1 in every 100th SNP for an average spacing of 689‐bp distance between SNPs.

### 2.6 Admixture and principal components analysis (PCA)

For admixture analysis, we used the program NGSadmix (Skotte et al., 2013), which uses a genotype‐likelihood approach that factors in uncertainty associated with next‐generation sequencing and has been shown to have good performance even with low‐coverage data. We ran NGSadmix using the BEAGLE genotype likelihood files created by ANGSD with *K* values ranging from 2 to 6 (*K* = number of assumed genetic clusters). We included only SNPs that were present in at least 94% of all individuals and had a minimum minor allele frequency of 5%. Here, we focus on the results from *K* = 4 genetic clusters because we are interested in assessing the contributions of the four ancestral lineages (A, M, C, and O) historically imported into the Americas. However, we also report admixture results from *K* = 2–6 in Figure S1. We used R (R Core Team, 2014) to graph admixture estimates. We used PCAngsd (Korneliussen et al., 2014) to conduct a principal components analysis of all SNPs, and graphed the resulting PCA with R (R Core Team, 2014).

### 2.7 Mitochondrial sequence assembly and phylogenetic analysis

Filtered reads of all 60 New World honey bees were aligned to a mitochondrial reference genome from an individual of subspecies *A. m. ligustica* sequenced by Crozier and Crozier (1993) using the BWA v0.7.12 bwa mem algorithm under default settings (Li & Durbin, 2009). We then called variants using samtools v1.10v (mpileup function) and used bcftools v1.10.2 (Li, 2011; Li & Durbin, 2009; Li et al., 2009) to extract the consensus sequence and convert to FASTQ with the vcfutils.pl script. We downloaded 12 previously assembled full mitochondrial sequences from *A*. *mellifera* subspecies representing all four major lineages from NCBI to compare with our samples (listed in Table 2).

**TABLE 2:** Mean percentage (SE) of genomic contributions from the four major honey bee lineages in each sampled population (*n* = 15 bee genomes per sample)

Clade ancestry	San Diego, CA	Mexico	Costa Rica	Panamá
African (A)	37.5% ± 1.12%	76.7% ± 0.61%	85.4% ± 0.07%	89.6% ± 0.17%
Western European (M)	19.1% ± 0.41%	14.9% ± 0.36%	10.5% ± 0.42%	10.3% ± 0.12%
Eastern European (C)	34.8% ± 1.14%	8.07% ± 0.34%	4.05% ± 0.03%	0.09% ± 0.01%
Middle Eastern (O)	8.51% ± 0.24%	0.45% ± 0.33%	0.00% ± 1.9e−21%	0.00% ± 6.72e−21%

FASTQ files of mitochondrial sequences from all 73 honey bees (13 reference honey bees and 60 AHB samples) were aligned using MAFFT (Katoh et al., 2019), on the XSEDE via Cipres 2.0 Science Gateway. We used MEGAX (Kumar et al., 2018) and complete deletion of gaps and missing data to create a neighbor‐joining phylogeny under a Kimura 2‐parameter model to compute evolutionary distances. We then ran 2000 bootstrap replicates to estimate confidence in the resulting phylogeny.

### 2.8 Measures of genetic diversity

To assess allelic diversity, we calculated estimates of both pairwise theta ($\hat{\theta }$_π_), based on the number of mean pairwise differences between sequences, and Watterson's theta ($\hat{\theta }$ *_w_*), based on the measure of segregating sites for each sampled and reference population using ANGSD v.928 (Korneliussen et al., 2014). Our reference populations were created by including honey bees from both the Wallberg et al. (2014) and Harpur et al. (2014) sequencing projects. However, the Middle Eastern (Clade O) population contained only honey bees from Wallberg et al. (2014) because Harpur et al. (2014) did not sequence bees from this lineage. We additionally calculated genetic diversity measures for each reference population, separated by sequencing project, to ensure that the different sequencing methods did not unduly influence our results (Table S3). Using only sites in which at least 50% of individuals in a population provided data, we estimated the folded site frequency spectrum (SFS) across the entire genome using the reference honey bee genome as the ancestral state. We then calculated and averaged thetas per site, including invariant sites, using ANGSD's realSFS program. To ensure that our diversity estimates were not overly affected by the difference in coverage between our reference and newly‐sequenced genomes, we calculated an additional measure of pairwise nucleotide diversity ($\hat{\theta }$ *_π_*) using only higher confidence SNPs with >5% minor allele frequency (MAF) in the total sample, following a pipeline described in Calfee et al. (2020). Using ANGSD, we first identified a set of SNPs with >5% minor allele frequency in the total sample and inferred the major and minor alleles at those SNPs using observed base counts (‐doMajorMinor 2 ‐doCounts 1 ‐doMaf 8 –minMAF 0.05). We excluded SNPs where more than half of individuals in the total sample did not have coverage. Using this list of SNPs (*n* = 5,666,586) as a reference, we calculated allele frequencies for each population based on observed base counts in ANGSD (‐doMajorMinor 3 ‐doCounts 1 ‐doMaf 8). From these population allele frequencies, we calculated the average pairwise diversity per SNP, correcting for small sample sizes. To account for invariant sites in our estimate of nucleotide diversity (*π*), we weighted our measure of *π* per‐SNP by the genome SNP density (total number of SNPs/total positions in the genome). For each measure of genome‐wide nucleotide diversity, we estimated standard errors using a block‐jackknife procedure, treating each chromosome as a block and recomputing nucleotide diversity with sequential exclusion of each chromosome.

## 3 RESULTS

### 3.1 Global genomic ancestry in Africanized honey bee samples

**FIGURE 1:**
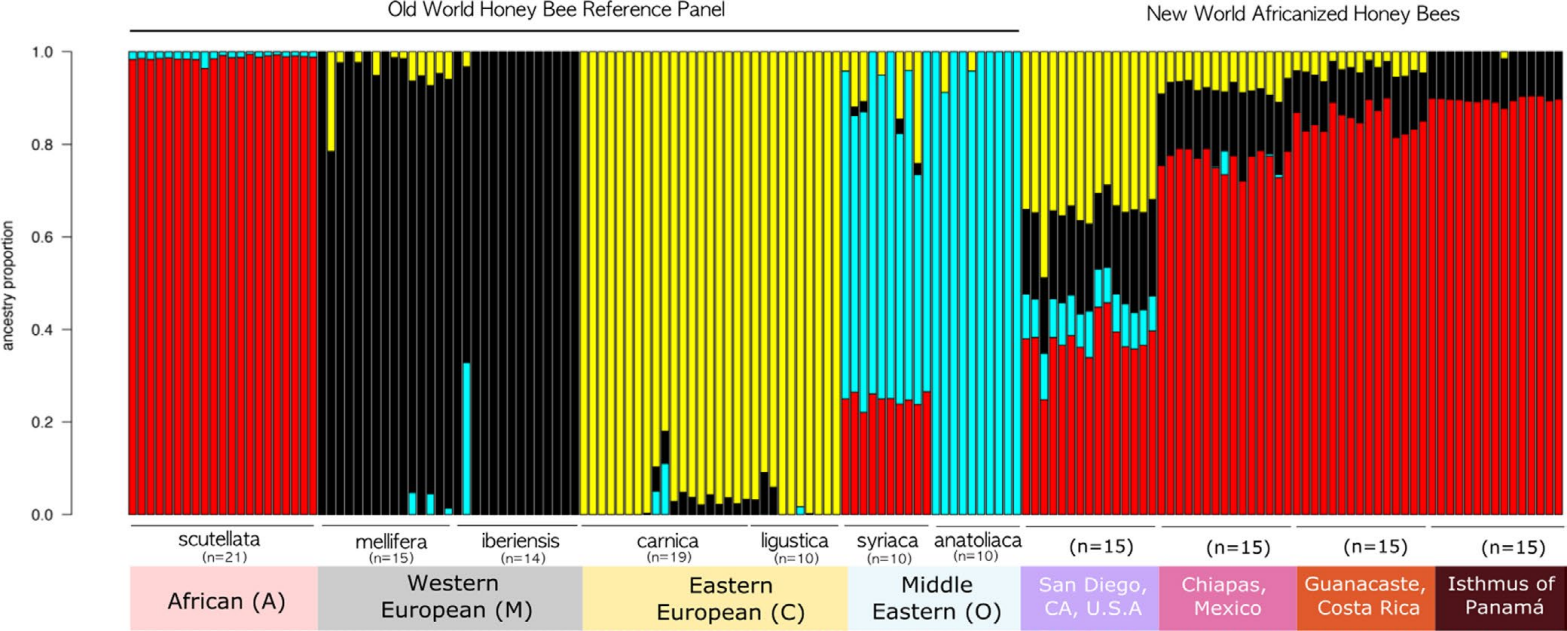
NGSadmix barplot of ancestry. Each vertical bar is one honey bee genome and colors represent the estimated proportion of ancestry derived from each genetic cluster (*K* = 4). The 99 reference genomes belonging to the four major evolutionary lineages of *Apis mellifera* (A, M, C, O) are grouped and labeled beginning with the African clade. The 60 admixed AHB genomes are arranged north to south by geographic origin, beginning with San Diego, CA and followed by the honey bees from Mexico, Costa Rica, and Panamá

At *K* = 4 clustering, the 99 reference honey bees from Wallberg et al. (2014) and Harpur et al. (2014) resolve into four groups representing the four recognized honey bee lineages (A, C, M & O), largely agreeing with previous genomic analyses (Chen et al., 2016; Cridland, Tsutsui, et al., 2017; Han et al., 2012; Nelson et al., 2017; Wallberg et al., 2014; Whitfield et al., 2006). Generally, there is limited evidence of admixture between these groups (Figure 1). However, honey bees of the subspecies *A. m. syriaca* (Clade O) are an exception, with ~20% of its ancestry attributed to the African clade, consistent with results found by Wallberg et al. (2014). Two individuals from the Western clade (M) (one from subspecies *A. m. mellifera* and one from subspecies *A. m. iberiensis*) showed significant ancestry from other clades (Clades C and O, respectively), a finding also consistent with Wallberg et al. (2014) (Figure 1). We also observed a small proportion (~1%) of Middle Eastern (O) ancestry across all the sampled honey bees from the African lineage *A. m. scutellata*. For results using different clustering values (*K* = 2–6), see Figure S1.

The nuclear genomes of honey bees from Central America and Mexico were heavily Africanized. Honey bees from the isthmus of Panamá averaged 89% (SE 0.17%) African (A) ancestry with the remaining 11% (SE 0.12%) of their genomes derived from the Western European (M) lineage. Honey bees from Guanacaste, Costa Rica, averaged 85% (SE 0.07%) African (A), 11% (SE 0.42%) Western (M) and 4% (SE 0.03%) Eastern European (C). In Chiapas, Mexico, honey bees averaged 77% (SE 0.61%) African (A), 15% (SE 0.36%) Western (M) and 8% (SE 0.34%) Eastern European (C) (Figure 1, Table 2).

In contrast to the honey bees of Central America and Mexico, genomes of all 15 honeybees sampled from San Diego (California, U.S.A.) exhibited a diverse admixture of all four major clades (A, M, C, and O). Ancestry of San Diego bees averaged 37% (SE 1.2%) African (A), 19% (SE 0.41%) Western European (M), 35% (SE 1.1%) Eastern European (C) and 9% (SE 0.22%) Middle Eastern (O) (Figure 1). African (A) ancestry of San Diego bees was far lower than that found in bees from any of the other sampled sites and contributions from the Eastern European (C) lineage were higher than all other populations sampled. All San Diego bees possessed substantial Middle Eastern (O) ancestry while all other sites sampled had negligible or no ancestry from this clade (Figure 1, Table 2).

### 3.2 Principal component analysis (PCA)

**FIGURE 2:**
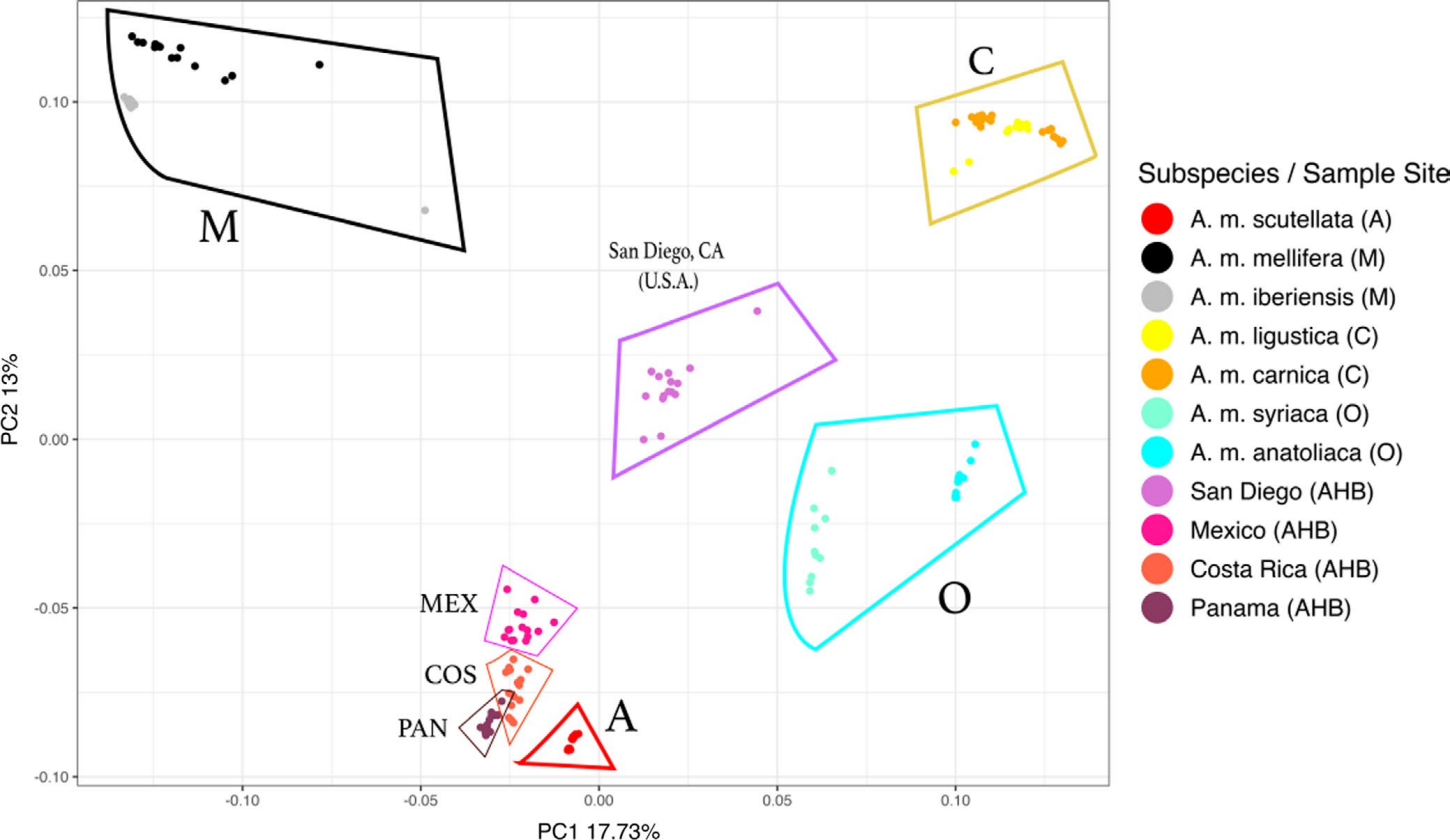
Principal Component Analysis (PCA) of the 99 reference honey bees and 60 admixed honey bee genomes

The principal components analysis of the 99 reference honey bees representing the four major honey bee clades (A, M, C, O) and the 60 honey bees we sampled from Panamá to San Diego separated populations by clade and sampling site (Figure 2). The ancestral honey bee lineages were widely separated from each other on the first two principal component axes. Bees from the four sampled sites (Panamá; Costa Rica; Mexico; San Diego, CA, U.S.A.) separated into distinct clusters with the exception of partial overlap among the bees from Panamá and Costa Rica. Bees from Mexico, Costa Rica, and Panamá clustered near African (A clade) honey bees. San Diego bees formed a more distant cluster relative to bees from Mexico, Costa Rica, and Panamá, falling more equidistantly between the A, M, C, and O groups, consistent with their ancestry drawing substantially from all four groups.

### 3.3 Mitochondrial ancestry in Africanized honey bee samples

**FIGURE 3:**
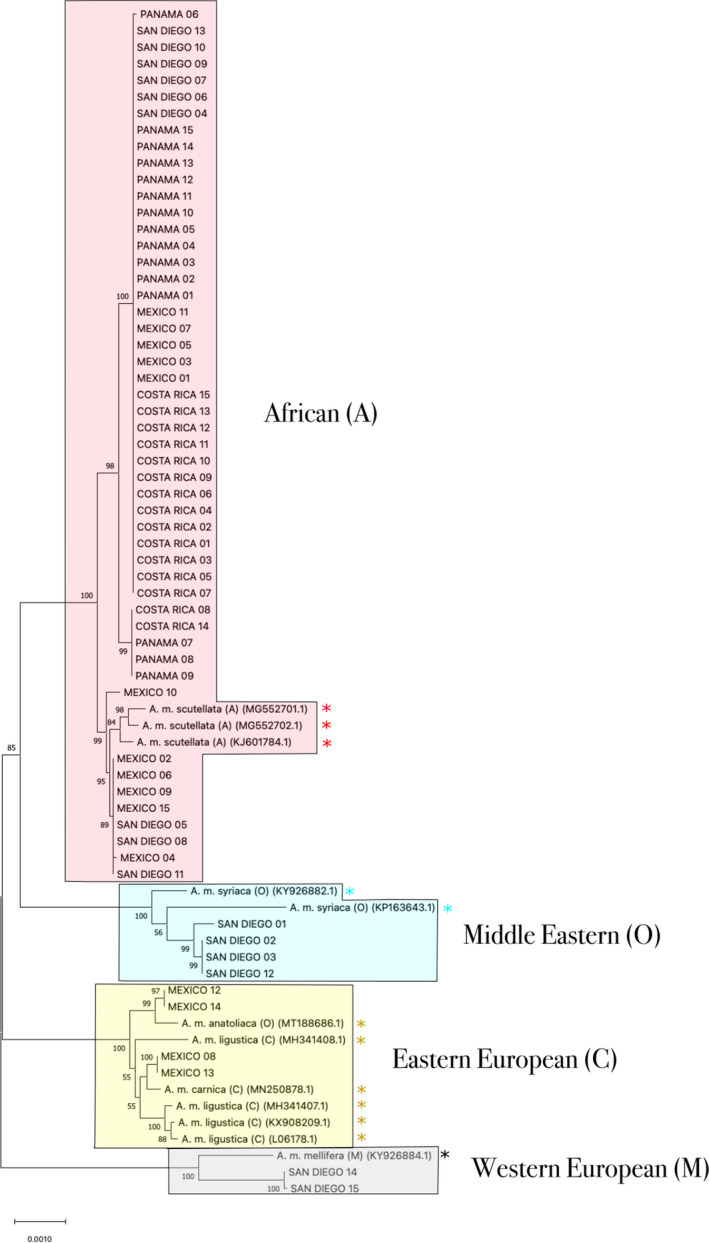
Midpoint‐rooted neighbor joining phylogeny constructed from the mitochondrial genomes of 60 admixed honey bees collected from San Diego, Mexico, Costa Rica, and Panamá (*n* = 15, per population) and 12 reference mitochondrial sequences obtained from NCBI: *A. m. mellifera* (*n* = 1), *A. m. syriaca* (*n* = 2), *A. m. carnica* (*n* = 1), *A. m. scutellata* (*n* = 3), *A. m. ligustica* (*n* = 4), and *A. m. anatoliaca* (*n* = 1). NCBI mitochondrial sequences are denoted by an asterisk (*). Values on each node represent the percent bootstrap support (*n* = 2000 bootstraps)

Each mitochondrial sequence from New World honey bees grouped strongly with reference mitochondria from one of the four ancestral lineages (A, M, C, O) in a midpoint rooted phylogeny (Figure 3, Table 3). Notably, mitochondrial sequences from subspecies *A. m. anatoliaca* (Clade O) grouped loosely with subspecies *A. m. ligustica* and *A. m. carnica* (both C). *A. m. anatoliaca* has previously been shown to possess C type mitochondria although it remains characterized as an O clade honey bee due to similarities of morphological and nuclear markers (Palmer et al., 2000; Smith et al., 1997; Wallberg et al., 2014). A mitochondrial phylogeny rooted with *A*.* cerana*, *A*.* florea*, and *A*. *dorsata* is presented in Figure S2.

**TABLE 3:** Whole mitochondrial sequences representing A/C/M/O honey bee clades downloaded from NCBI and used in mtDNA haplotype analysis

GenBank accession number	Subspecies	Clade
KJ601784.1	*A. m. scutellata*	A
MG552702.1	*A. m. scutellata*	A
MG552701.1	*A. m. scutellata*	A
KY926884.1	*A. m. mellifera*	M
MN250878.1	*A. m. carnica*	C
KX908209.1	*A. m. ligustica*	C
MH341408.1	*A. m. ligustica*	C
MH341407.1	*A. m. ligustica*	C
L06178.1	*A. m. ligustica*	C
MT188686.1	*A. m. anatoliaca*	O
KP163643.1	*A. m. syriaca*	O
KY926882.1	*A. m. syriaca*	O

### 3.4 Genetic diversity

All four sampled AHB populations have similar levels of genetic diversity for all three estimators which use both genome‐wide sites and a smaller number of high‐quality SNPs (Table 5). Genetic diversity estimates in the admixed AHB populations are consistently higher than those estimated for the European and Middle Eastern populations (clades M, C, and O) (Table 5). The admixed populations have similar genetic diversity as African honey bees (Clade A) for estimators calculated from genome‐wide sites. In contrast, the pairwise estimation of genetic diversity for admixed populations exceeds the genetic diversity of African honey bees when using only high‐quality SNPs in the analysis. Among ancestral lineages, for all measures the African lineage is the most diverse, followed by the Middle Eastern (O) lineage, the Western European (M) lineage and lastly, the Eastern European lineage (C).

## 4 DISCUSSION

Africanized honey bee (AHB) populations exhibit distinct genomic admixture profiles across their Central and North American range with African ancestry decreasing with increasing latitude (Figures 1 and 2; Table 2). Despite considerable differences in ancestral composition between populations, each sampled region exhibits little variation amongst individual honey bees. This is true whether honey bees were sampled over many tens of kilometers (Isthmus of Panama, San Diego, California) or from geographically restricted sampling points (Chiapas, Mexico and Guanacaste, Costa Rica). At the scales sampled, AHB populations appear to be well‐mixed hybrid swarms. Honey bees from Panama, Costa Rica, and Mexico all possess substantial amounts of African ancestry (76%–89%), similar to that reported in Brazil (Nelson et al., 2017; Wallberg et al., 2014). This extensive Africanization is perhaps reflective of the longstanding presence of AHB in these regions and that little is done to prevent Africanization of managed hives for both agricultural and hobbyist use. Beekeepers in Central America and Mexico have adapted to working with the AHB and gene flow between feral and managed hives is largely uninhibited (Guzman‐Novoa & Page, 1999; Ratnieks & Visscher, 1996).

The substantial amount of Eastern European (C) ancestry persisting in the honey bees sampled from Mexico suggests that insufficient time may have passed since the arrival of AHB for honey bees to reach the high levels of African ancestry seen in lower latitudes. However, AHB first arrived in southern Mexico in the late 1980s, and studies have shown that levels of African ancestry can reach high, apparently stable, levels in less than a decade (Pinto et al., 2005). Alternatively, the substantial European honey bee (EHB) population that existed throughout Mexico prior to AHB arrival might have served as a genetic buffer, allowing for the persistence of C ancestry despite ample time since contact with AHB (Clarke et al., 2002). Beekeeping with C‐lineage honey bees was widespread across Mexico prior to the arrival of AHB, with an estimated 1.5 million managed colonies present throughout the country and Mexico remains one of the largest exporters of honey on the global market (Guoda et al., 2001; Winston, 1979). In contrast, Costa Rica and Panamá both had modest managed beekeeping activity prior to AHB arrival, and feral EHB colonies were quite rare, particularly in the rainy lowlands (Lobo, 1995; Roubik & Boreham, 1990). Additionally, many beekeepers in Central America abandoned the trade after AHB arrival (van Veen et al., 1998). Thus, AHB likely encountered a much smaller population of EHB in Central America than in Mexico, allowing for a rapid and extensive Africanization of the honey bee gene pool.

In striking contrast to the honey bees from Mexico and Central America, African ancestry in honey bees collected in San Diego County, California (U.S.A.) is relatively low ($\bar{x}$ = 37.5% ± 1.12%) and San Diego bees feature substantial genomic contributions from all four major honey bee lineages (Figures 1 and 2). Eastern European (C) ancestry ($\bar{x}$ = 34.8% ± 1.14%) in San Diego honey bees is substantially higher than the other three sampled sites while the contribution of the Western European (M) lineage ($\bar{x}$ = 19.1% ± 0.41%) is also somewhat elevated (Figure 1, Table 2). Honey bees from San Diego also have lower African genomic content in comparison to those from Texas and Arizona (~75% A) (Bozek et al., 2018; Whitfield et al., 2006).

Notably, all honey bees from the San Diego sample possessed considerable Middle Eastern (O) ancestry ($\bar{x}$ = 8.5% ± 0.24%). Honey bees from Middle Eastern lineages were imported to the United States during the last two decades of the 19th century after which time these limited importations stopped (Magnus & Szalanski, 2010 and references therein; Carpenter & Harpur, 2021). Nevertheless, surveys of feral honey bees in the United States have reported the continued presence of O‐clade mitochondria (Kono & Kohn, 2015; Magnus & Szalanski, 2010), and Whitfield et al. (2006) found evidence of some O nuclear genomic content in AHB in Texas. Our findings concerning relatively low levels of African ancestry in San Diego largely agree with other recent genomic studies of feral honey bees in southern California (Calfee et al., 2020; Cridland, Ramirez, et al., 2017). However, this is the first assessment of Middle Eastern (O) ancestry in southern California honey bees.

Why is African genomic content in southern California bees much lower than elsewhere? We explore three hypotheses that might account for this. First, models built from climate data fitted to the southern AHB range limit predict that colder winter weather plays a considerable role in halting AHB expansion. (Harrison et al., 2006; Southwick et al., 1990; Taylor & Spivak, 1984). Nearer the northern (California, USA) and southern (Buenos Aires, Argentina) range limits of AHB, African ancestry is notably reduced in favor of European ancestry (Calfee et al., 2020). Western San Diego County has a mild Mediterranean climate featuring dry summers with a mean high temperature of 25°C (August) and mild winters with a mean minimum of 8°C (January) (NOAA–National Weather Service Forecast Office). South Texas, where African genomic content is much higher than in San Diego, has a hot and humid climate, on average reaching 35°C in the hottest summer month but possesses similarly cool winters to those in San Diego, reaching an average minimum of 7°C in January) (Rangel et al., 2016; NOAA–National Weather Service Forecast Office). Thus, while climate is likely important in limiting the penetrance of African genomic material, simple measures of winter cold temperatures are unlikely to be the only determining factor.

Second, gene flow from managed European honey bee populations could restrain the introgression of genes of African origin in San Diego County. In the United States, AHB are generally considered unfit for apiculture and commercial agriculture due to undesirable characteristics such as a higher propensity to sting and to abandon their nests (reviewed in Schneider et al., 2004). The desired lineages of European clades are actively maintained via consistent requeening of colonies with mated queens of European origin (Schiff & Sheppard, 1995, 1996). However, such beekeeping practices have failed to noticeably inhibit the introgression of high levels of African genes into feral Texas and Arizona bee populations (Bozek et al., 2018; Pinto et al., 2005; Whitfield et al., 2006). One potential mitigating factor preventing excessive Africanization of San Diego honey bees may be the large agricultural presence in the county with ~230,000 acres of planted crops, many of which (e.g., avocados and citrus) use honey bees for pollination services (San Diego County Crop Statistics Annual Report, 2019). Gene flow from high‐density European managed hives could counter Africanization. However, genetic swamping by managed honey bees would require that a substantial fraction of the honey bees in San Diego County come from managed, genetically European hives. Our finding that all 15 foraging workers examined here had substantial African and Middle Eastern ancestry—lineages not used in managed colonies—argues against this. This finding is consistent with the hypothesis, supported by previous (Kono & Kohn, 2015) and current mitochondrial data, that most bees foraging in San Diego County, whether in urban or nonagricultural rural settings, derive from feral, Africanized colonies.

Finally, insufficient time may have elapsed since the introduction of the AHB to San Diego County for African ancestry to reach levels comparable to those seen elsewhere in the southwestern U.S. (Pinto et al., 2005; Whitfield et al., 2006). AHB arrived in San Diego County in 1994 and our bees were sampled more than two decades later, suggesting either that Africanization is taking much longer than in Texas, or differences in conditions in San Diego relative to Texas lead to reduced penetration of the African genome.

Notably, all four sampled regions report a significant amount of Western European (M) ancestry. Studies that have tracked the process of Africanization elsewhere have shown that African genetic material largely or completely replaces genomic content from the Eastern European (C) lineage, while the contribution from the M lineage to genomes of AHB remains substantial and is never completely eliminated (Clarke et al., 2002; Cridland, Ramirez, et al., 2017; Nelson et al., 2017; Pinto et al., 2005; Whitfield et al., 2006). All of our sampled honey bee genomes from San Diego to Panamá possess moderate levels of M ancestry while C ancestry content declines precipitously from north to south and is nearly totally absent in samples from Costa Rica and Panamá. This pattern suggests that the M‐lineage content that persists in highly Africanized populations may be selected for while C‐lineage content is selected against except where A‐lineage contribution declines at higher latitudes (Nelson et al., 2017; Whitfield et al., 2006). Previous studies have identified some regions of Western European (M) ancestry that appear to be under positive selection, in particular a region on chromosome 13, which is associated with a QTL for worker ovary size (Calfee et al., 2020; Nelson et al., 2017). In addition, genes of both M and A ancestry appear to underlie nest defense behavior in AHB (Harpur et al., 2020). Further work is needed to determine whether these regions of M ancestry are under selection in our sampled populations. Alternatively, small amounts of M ancestry may be hitchhiking within predominantly African genomes.

Mitochondrial analysis of our four sampled populations is largely consistent with findings from nuclear genomes (Figure 3; Table 4). All bees sampled from Panamá and Costa Rica, where nuclear genomes were predominantly African, carried mitochondria of African origin. In Mexico, the majority of honey bees carried the A mitotype while a few carried mitochondria of the C lineage. San Diego honey bees harbor all four mitochondrial lineages. While C‐lineage mitochondria were absent in our current sample of 15 bees, Kono and Kohn (2015) assayed a larger sample and found mitotypes representing all four clades, with the African mitotype the most frequent (65%) and mitochondria from the other three lineages present in similar proportions. Failure to uncover any mitochondria from the C lineage in the present study likely resulted from the limited number of honey bees sampled.

**TABLE 4:** Number of honey bees sampled from each admixed population (San Diego (CA), Mexico, Costa Rica, Panamá) found to carry mitochondria from each of the four clades (A, M, C, O)

	San Diego, CA	Mexico	Costa Rica	Panamá
African (A)	9	11	15	15
Western European (M)	2	0	0	0
Eastern European (C)	0	4	0	0
Middle Eastern (O)	4	0	0	0

**TABLE 5:** Genetic diversity measures (mean ± SE) for admixed and reference populations

Population	Pairwise estimator ($\hat{\theta }$ *_π_*)	Pairwise estimator ($\hat{\theta }$ *_π_*) using called SNPs only (minor allele frequency >0.05)	Watterson's estimator ($\hat{\theta }$ *_w_*)
African (A)	0.0100 ± 0.0018	0.0047 ± 9.205e−05	0.0152 ± 0.0012
Western European (M)	0.0047 ± 0.0007	0.0028 ± 3.503e−05	0.0049 ± 0.0006
Eastern European (C)	0.0036 ± 0.0003	0.0023 ± 2.343e−05	0.0042 ± 0.0004
Middle Eastern (O)	0.0059 ± 0.0005	0.0037 ± 5.569e−05	0.0062 ± 0.0004
San Diego, CA, U.S.A.	0.0096 ± 0.0018	0.0061 ± 8.863e−05	0.0108 ± 0.0020
Chiapas, Mexico	0.0109 ± 0.0023	0.0063 ± 0.0001	0.0124 ± 0.0023
Guanacaste, Costa Rica	0.0105 ± 0.0024	0.0061 ± 0.0001	0.0110 ± 0.0021
Isthmus of Panamá	0.0106 ± 0.0024	0.0060 ± 0.0001	0.0114 ± 0.0020

Admixed populations from the four sampled sites report similar high levels of genetic diversity and these levels are higher than genetic diversity measures from Eastern European (C), Western European (M), and Middle Eastern (O) reference populations. Additionally, depending on the estimator, the genetic diversity of admixed populations exceeds or equals that of the African clade, the honey bee lineage previously found to have the highest genetic diversity (Calfee et al., 2020; Espregueira Themudo et al., 2020; Harpur et al., 2012; Wallberg et al., 2014). The high level of genetic diversity of admixed populations likely results from both substantial contributions from the genetically diverse African lineage in addition to admixture bringing together variation found among ancestral lineages (Harpur et al., 2012). For San Diego bees, the effect of admixture from multiple ancestral lineages appears to raise their genetic diversity to levels not different than those found in A lineage reference bees or in AHB populations with much higher proportions of A ancestry.

Wallberg et al. (2014) employed a SNP‐based measure to calculate Watterson's estimator and then divided the measure by total sites in the genome in order to obtain a per‐bp estimate. Our pairwise estimator was calculated in a similar fashion and gave similar, though slightly lower, values. Two of our genetic diversity estimates (genome‐wide pairwise and Watterson's estimators) were calculated from the majority of sites in the genome, including both high‐quality SNPs and other variable and invariable sites. Those methods resulted in higher estimates of genetic diversity. However, the rank order of our diversity estimates among reference populations, with the A lineage having considerably higher diversity than the O lineage, followed by M and then C is consistent with previous studies (Espregueira Themudo et al., 2020; Harpur et al., 2012; Wallberg et al., 2014).

We find that the honey bees of the subspecies *A. m. syriaca* are substantially (~23%) admixed with the African lineage, consistent with the analysis of Wallberg et al. (2014). Cridland, Tsutsui, et al. (2017) ascribed a similar amount of admixture into the *A. m. syriaca* bees from a population of bees from the Arabian Peninsula (termed Clade Y). We have not included bees from the Y lineage in our analysis because they are not known to have been introduced to the American continents (Carpenter & Harpur, 2021; Ruttner, 1988). It is possible that the admixture in *A. m. syriaca* reported here and in Wallberg et al. (2014) represents intermixing with bees from the Arabian Peninsula rather than Africa.

Africanized honey bees in the New World represent one of the largest and best‐documented biological invasions resulting from human‐mediated hybridization. Feral European honey bee populations have been replaced by Africanized honey bees throughout most of the New World suggesting their genetic makeup provides strong ecological advantages except at higher latitudes. In San Diego County, feral AHB are super abundant, responsible for 75% of all floral visits to native plants and reaching greater dominance (>90% of all pollinator visits) on the most abundantly blooming species (Hung et al., 2018, 2019). This occurs in spite of carrying detrimental viral diseases at titers similar to those found in managed bees, suggesting they can resist negative viral affects for which managed hives receive mitigating treatments (Geffre et al., 2021). Future work to determine local genomic ancestry could investigate selection on genomic regions that consistently come from African versus European lineages. Such regions, and the genes they contain, are critical to understanding the genetic changes that underlie the ecological success of Africanized honey bees. Such analyses could also shed light on the locations and origins of genomic regions useful for breeding managed honey bees to be more resistant to factors currently harming the honey bee industry.

## CONFLICT OF INTEREST

Grants to J.R.K., R.S.B, D.Z., and T.L. supported the research. D.Z and J.R.K designed the project. D.Z. performed sampling, DNA preparation and data analysis. E.C., T. L. and J.P and J.R.K. aided in data analysis. D.Z. and J.R.K. were principal authors of the paper and all other authors reviewed and approved the final version of the manuscript.

## AUTHOR CONTRIBUTIONS

**Daniela Zarate:** Conceptualization (equal); Data curation (lead); Formal analysis (lead); Funding acquisition (lead); Methodology (lead); Project administration (lead); Visualization (lead); Writing – original draft (lead); Writing – review & editing (lead). **Thiago G. Lima:** Data curation (supporting); Funding acquisition (supporting); Methodology (supporting); Writing – review & editing (supporting). **Jude D. Poole:** Data curation (supporting); Formal analysis (supporting); Software (supporting). **Erin Calfee:** Formal analysis (supporting); Software (supporting); Visualization (supporting); Writing – review & editing (supporting). **Ronald S. Burton:** Funding acquisition (supporting); Methodology (supporting); Writing – review & editing (supporting). **Joshua R. Kohn:** Conceptualization (equal); Methodology (equal); Supervision (equal); Writing – original draft (equal); Writing – review & editing (equal).

## Supporting information

Supplementary Material

## Data Availability

All DNA sequence files are deposited and publicly available on the Dryad database (https://doi.org/10.5061/dryad.xwdbrv1ff).
