# Supplementary material for: Admixture in Africanized honey bees (Apis mellifera) from Panamá to San Diego, California (U.S.A.)
Source: Ecol Evol. 2022 Feb 14;12(2):e8580. doi: 10.1002/ece3.8580 (PMC8844128; doi:10.1002/ece3.8580)
Supplement: Supplementary file 1 — Supplementary Material [file ECE3-12-e8580-s001.docx]

**SUPPLEMENTARY FIGURES**


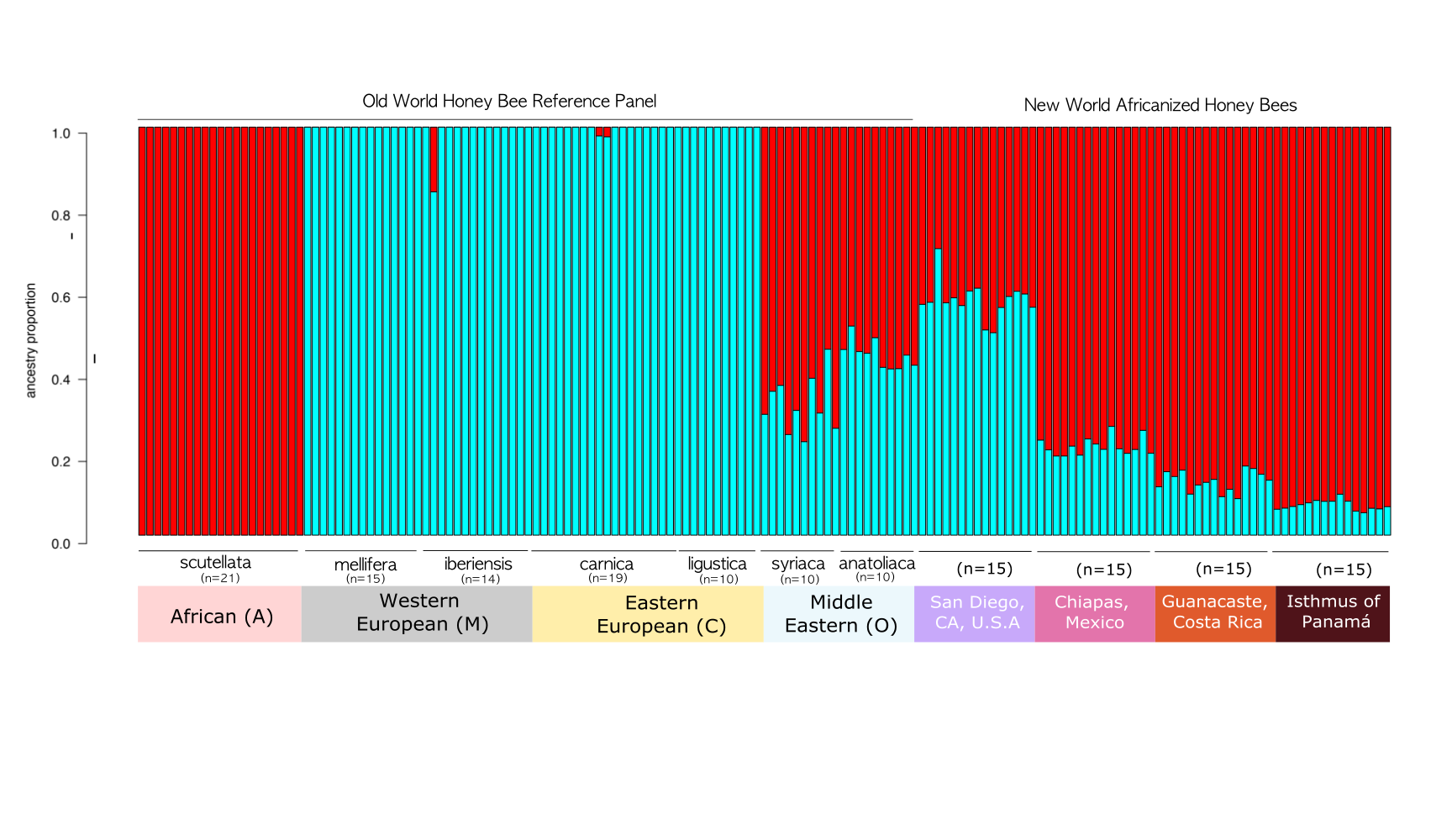


K=2


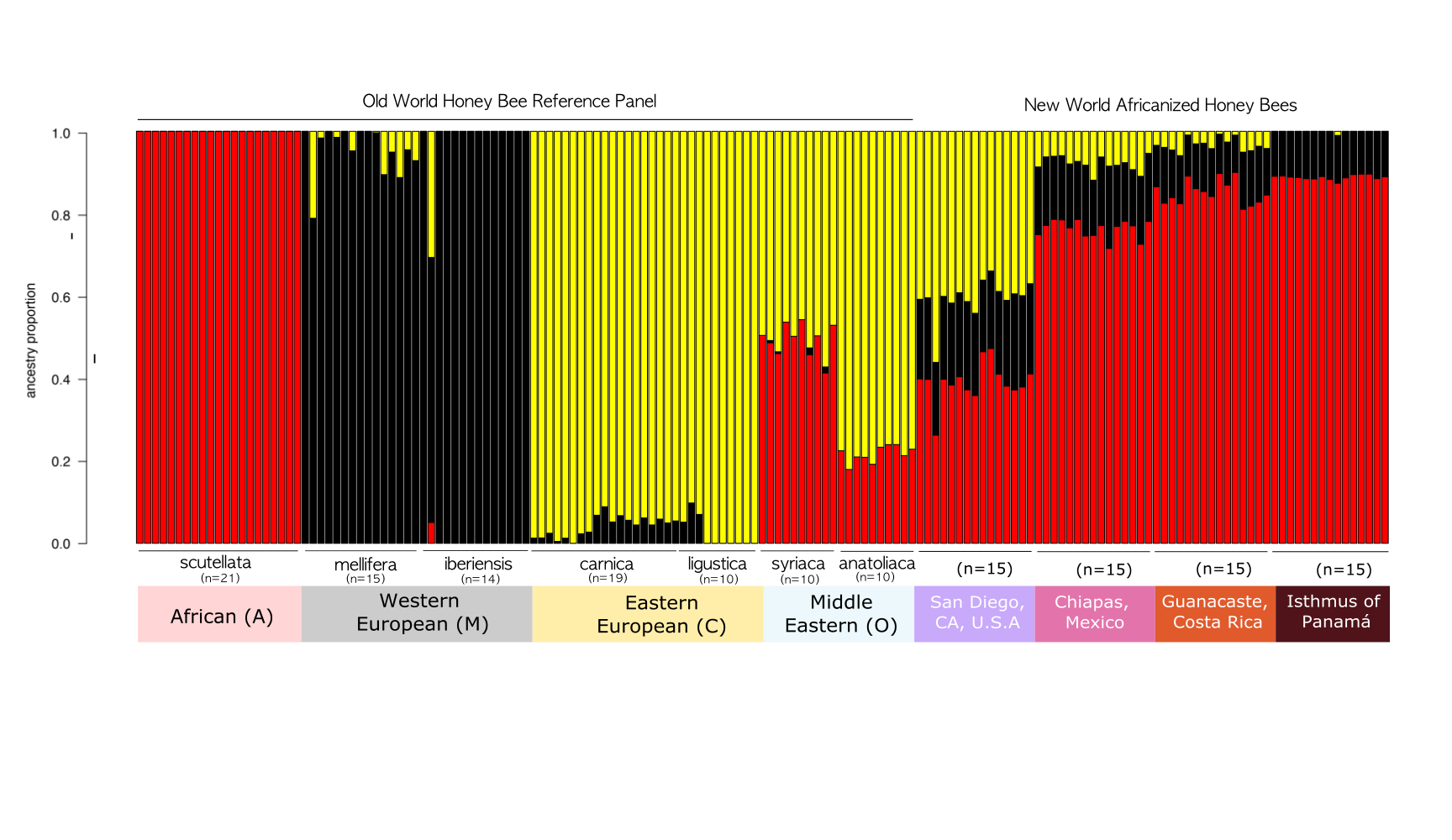


K=3


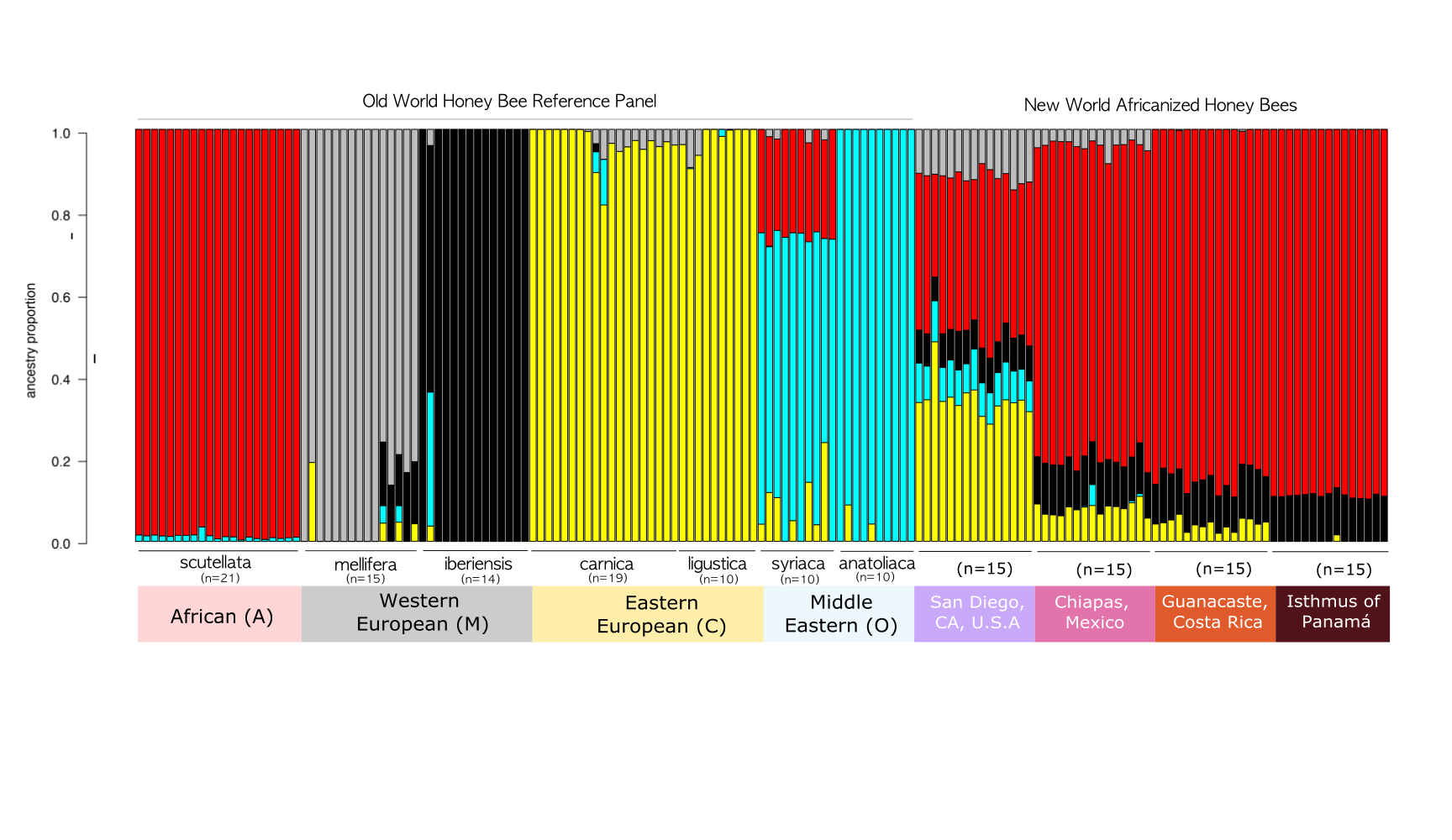


K=5


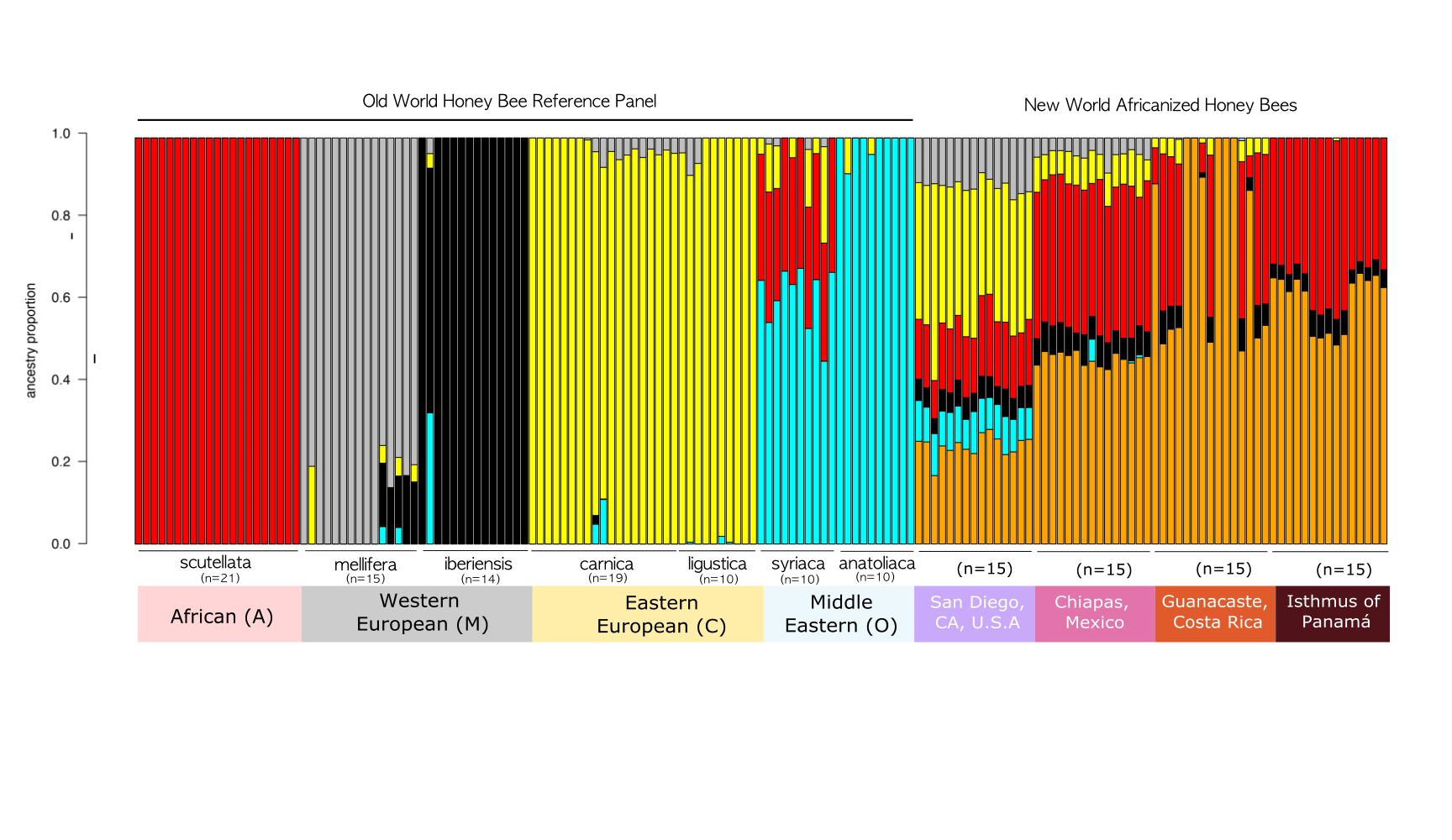


K=6

Figure S1. NGSadmix barplot of ancestry (K=2,3,5,6). Each vertical bar is one honey bee genome and colors represent the estimated proportion of ancestry derived from each genetic cluster (K= 2-6). The 70 reference genomes belonging to the four major evolutionary lineages of *Apis mellifera* (A, M, C, O) are grouped and labeled beginning with the African clade. The 60 admixed AHB genomes are arranged north to south by geographic origin, beginning with San Diego, CA and followed by the honey bees from Mexico, Costa Rica, and Panamá.


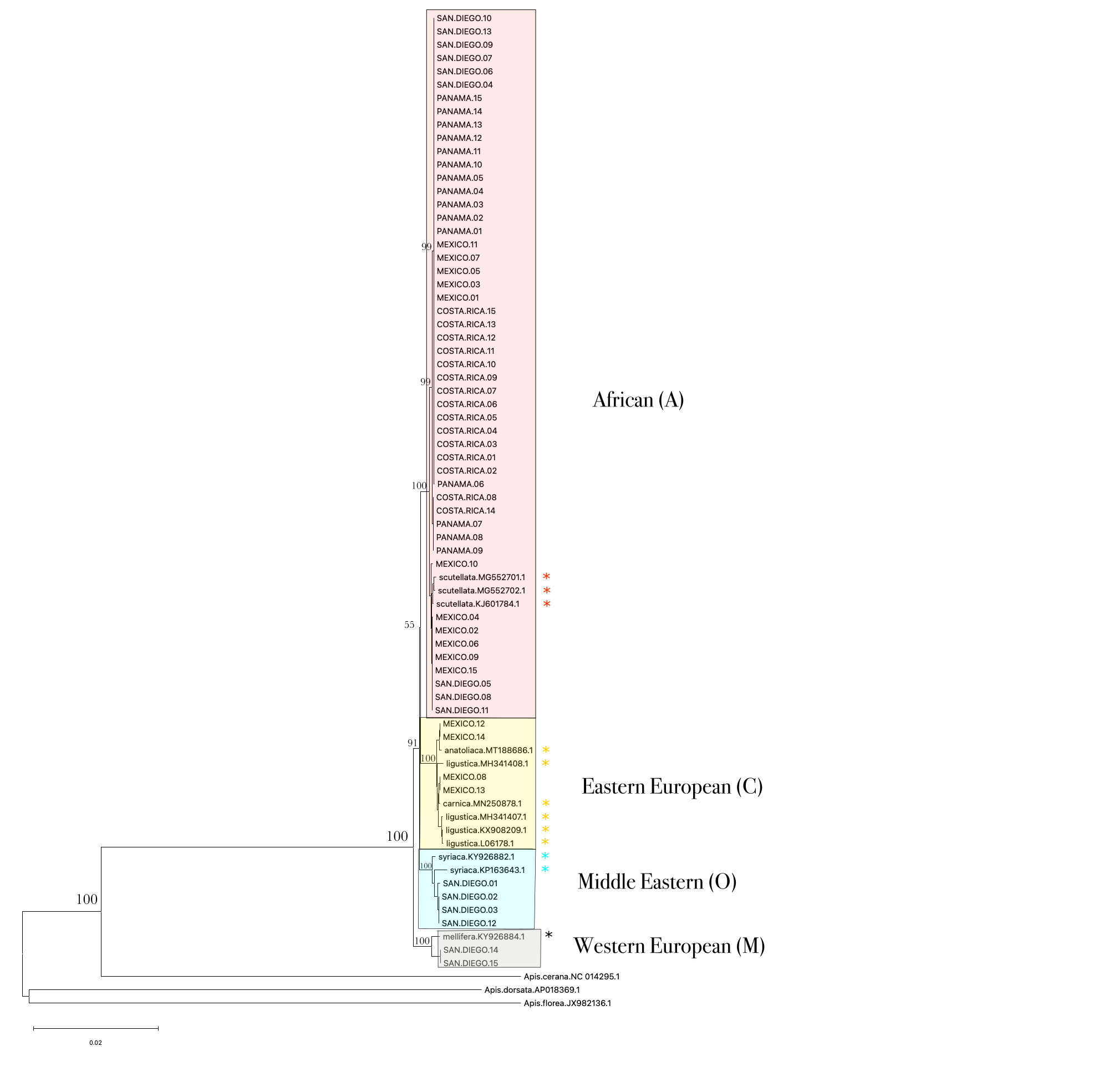


Figure S2: Neighbor joining phylogeny constructed from the mitochondrial genomes of 60 admixed honey bees collected from San Diego, Mexico, Costa Rica, and Panamá (n=15, per population) and 12 reference mitochondrial sequences obtained from NCBI: *A. m. mellifera* (n=1)*, A. m. syriaca* (n=2)*, A. m. carnica* (n=1)*, A. m. scutellata* (n=3)*, A. m. ligustica* (n=4)*,* and *A. m. anatoliaca* (n=1). Values on each node represent the percent bootstrap support (n = 2000 bootstraps). Tree is rooted with outgroup species: *Apis cerana, Apis dorsata*, and *Apis florea*, obtained from NCBI.

Table S1: Accession numbers for all reference honey bees downloaded from Wallberg *et al.,* (2014) and Harpur *et al.,* (2014) for use in ancestry admixture analyses.

| Individual | Label | Accession | Sequencing Project |
| --- | --- | --- | --- |
| **African (A)** |  |  |  |
| Apis mellifera scutellata | B7 | SAMN02596307 | Wallberg *et al.,* (2014) |
| Apis mellifera scutellata | B6 | SAMN02596306 | Wallberg *et al.,* (2014) |
| Apis mellifera scutellata | B5 | SAMN02596305 | Wallberg *et al.,* (2014) |
| Apis mellifera scutellata | B4 | SAMN02596304 | Wallberg *et al.,* (2014) |
| Apis mellifera scutellata | JOZI12 | SAMN02596303 | Wallberg *et al.,* (2014) |
| Apis mellifera scutellata | G1 | SAMN02596302 | Wallberg *et al.,* (2014) |
| Apis mellifera scutellata | TR2 | SAMN02596301 | Wallberg *et al.,* (2014) |
| Apis mellifera scutellata | URS1 | SAMN02596300 | Wallberg *et al.,* (2014) |
| Apis mellifera scutellata | URS3 | SAMN02596299 | Wallberg *et al.,* (2014) |
| Apis mellifera scutellata | URS4 | SAMN02596298 | Wallberg *et al.,* (2014) |
| Apis mellifera scutellata | S302 | SRX339526 | Harpur *et al.,* (2014) |
| Apis mellifera scutellata | S301 | SRX339525 | Harpur *et al.,* (2014) |
| Apis mellifera scutellata | S300 | SRX339524 | Harpur *et al.,* (2014) |
| Apis mellifera scutellata | S299 | SRX339523 | Harpur *et al.,* (2014) |
| Apis mellifera scutellata | S296 | SRX339522 | Harpur *et al.,* (2014) |
| Apis mellifera scutellata | S290 | SRX339521 | Harpur *et al.,* (2014) |
| Apis mellifera scutellata | S286 | SRX339520 | Harpur *et al.,* (2014) |
| Apis mellifera scutellata | S285 | SRX339507 | Harpur *et al.,* (2014) |
| Apis mellifera scutellata | S284 | SRX339506 | Harpur *et al.,* (2014) |
| Apis mellifera scutellata | S280 | SRX339505 | Harpur *et al.,* (2014) |
| Apis mellifera scutellata | S279 | SRX339504 | Harpur *et al.,* (2014) |
| **Western European (M)** |  |  |  |
| Apis mellifera mellifera | Ho6 | SAMN02596423 | Wallberg *et al.,* (2014) |
| Apis mellifera mellifera | Ho4 | SAMN02596422 | Wallberg *et al.,* (2014) |
| Apis mellifera mellifera | K5 | SAMN02596421 | Wallberg *et al.,* (2014) |
| Apis mellifera mellifera | G8 | SAMN02596420 | Wallberg *et al.,* (2014) |
| Apis mellifera mellifera | S4 | SAMN02596419 | Wallberg *et al.,* (2014) |
| Apis mellifera mellifera | S3 | SAMN02596418 | Wallberg *et al.,* (2014) |
| Apis mellifera mellifera | Ho7 | SAMN02596417 | Wallberg *et al.,* (2014) |
| Apis mellifera mellifera | Ho8 | SAMN02596416 | Wallberg *et al.,* (2014) |
| Apis mellifera mellifera | K9 | SAMN02596415 | Wallberg *et al.,* (2014) |
| Apis mellifera mellifera | S1 | SAMN02596414 | Wallberg *et al.,* (2014) |
| Apis mellifera iberiensis | 272A | SAMN02596387 | Wallberg *et al.,* (2014) |
| Apis mellifera iberiensis | 259B | SAMN02596386 | Wallberg *et al.,* (2014) |
| Apis mellifera iberiensis | 224B | SAMN02596385 | Wallberg *et al.,* (2014) |
| Apis mellifera iberiensis | 222A | SAMN02596384 | Wallberg *et al.,* (2014) |
| Apis mellifera iberiensis | 205A | SAMN02596383 | Wallberg *et al.,* (2014) |
| Apis mellifera iberiensis | 29B | SAMN02596382 | Wallberg *et al.,* (2014) |
| Apis mellifera iberiensis | 25A | SAMN02596381 | Wallberg *et al.,* (2014) |
| Apis mellifera iberiensis | 080B | SAMN02596380 | Wallberg *et al.,* (2014) |
| Apis mellifera iberiensis | 010A | SAMN02596379 | Wallberg *et al.,* (2014) |
| Apis mellifera iberiensis | 7B | SAMN02596378 | Wallberg *et al.,* (2014) |
| Apis mellifera mellifera | M217 | SRX339519 | Harpur *et al.,* (2014) |
| Apis mellifera mellifera | M207 | SRX339518 | Harpur *et al.,* (2014) |
| Apis mellifera mellifera | M256 | SRX339493 | Harpur *et al.,* (2014) |
| Apis mellifera mellifera | M248 | SRX339492 | Harpur *et al.,* (2014) |
| Apis mellifera iberiensis | M234 | SRX339491 | Harpur *et al.,* (2014) |
| Apis mellifera iberiensis | M233 | SRX339490 | Harpur *et al.,* (2014) |
| Apis mellifera iberiensis | M227 | SRX339489 | Harpur *et al.,* (2014) |
| Apis mellifera iberiensis | M226 | SRX339488 | Harpur *et al.,* (2014) |
| Apis mellifera iberiensis | M218 | SRX339487 | Harpur *et al.,* (2014) |
| **Eastern European (C)** |  |  |  |
| Apis mellifera carnica | CR10 | SAMN02596347 | Wallberg *et al.,* (2014) |
| Apis mellifera carnica | CR9 | SAMN02596346 | Wallberg *et al.,* (2014) |
| Apis mellifera carnica | CR8 | SAMN02596345 | Wallberg *et al.,* (2014) |
| Apis mellifera carnica | CR7 | SAMN02596344 | Wallberg *et al.,* (2014) |
| Apis mellifera carnica | CR6 | SAMN02596343 | Wallberg *et al.,* (2014) |
| Apis mellifera carnica | CR5 | SAMN02596342 | Wallberg *et al.,* (2014) |
| Apis mellifera carnica | CR4 | SAMN02596341 | Wallberg *et al.,* (2014) |
| Apis mellifera carnica | CR3 | SAMN02596339 | Wallberg *et al.,* (2014) |
| Apis mellifera carnica | CR2 | SAMN02596338 | Wallberg *et al.,* (2014) |
| Apis mellifera carnica | CR10 | SAMN02596337 | Wallberg *et al.,* (2014) |
| Apis mellifera ligustica | 12 | SAMN02596297 | Wallberg *et al.,* (2014) |
| Apis mellifera ligustica | 10 | SAMN02596296 | Wallberg *et al.,* (2014) |
| Apis mellifera ligustica | 8 | SAMN02596295 | Wallberg *et al.,* (2014) |
| Apis mellifera ligustica | 6 | SAMN02596294 | Wallberg *et al.,* (2014) |
| Apis mellifera ligustica | 5 | SAMN02596293 | Wallberg *et al.,* (2014) |
| Apis mellifera ligustica | 9 | SAMN02596292 | Wallberg *et al.,* (2014) |
| Apis mellifera ligustica | 11 | SAMN02596291 | Wallberg *et al.,* (2014) |
| Apis mellifera ligustica | 7 | SAMN02596290 | Wallberg *et al.,* (2014) |
| Apis mellifera ligustica | 2 | SAMN02596289 | Wallberg *et al.,* (2014) |
| Apis mellifera carnica | C273 | SRX339517 | Harpur *et al.,* (2014) |
| Apis mellifera carnica | C236 | SRX339516 | Harpur *et al.,* (2014) |
| Apis mellifera carnica | C235 | SRX339515 | Harpur *et al.,* (2014) |
| Apis mellifera carnica | C200 | SRX339514 | Harpur *et al.,* (2014) |
| Apis mellifera carnica | C199 | SRX339513 | Harpur *et al.,* (2014) |
| Apis mellifera carnica | C196 | SRX339512 | Harpur *et al.,* (2014) |
| Apis mellifera carnica | C195 | SRX339511 | Harpur *et al.,* (2014) |
| Apis mellifera carnica | C182 | SRX339510 | Harpur *et al.,* (2014) |
| Apis mellifera carnica | C181 | SRX339509 | Harpur *et al.,* (2014) |
| **Middle Eastern (O)** |  |  |  |
| Apis mellifera anatoliaca | AN12 | SAMN02596377 | Wallberg *et al.,* (2014) |
| Apis mellifera anatoliaca | AN11 | SAMN02596376 | Wallberg *et al.,* (2014) |
| Apis mellifera anatoliaca | AN8 | SAMN02596375 | Wallberg *et al.,* (2014) |
| Apis mellifera anatoliaca | AN7 | SAMN02596374 | Wallberg *et al.,* (2014) |
| Apis mellifera anatoliaca | AN6 | SAMN02596373 | Wallberg *et al.,* (2014) |
| Apis mellifera anatoliaca | AN5 | SAMN02596372 | Wallberg *et al.,* (2014) |
| Apis mellifera anatoliaca | AN3B | SAMN02596371 | Wallberg *et al.,* (2014) |
| Apis mellifera anatoliaca | AN1 | SAMN02596370 | Wallberg *et al.,* (2014) |
| Apis mellifera anatoliaca | A2B | SAMN02596369 | Wallberg *et al.,* (2014) |
| Apis mellifera anatoliaca | A3A | SAMN02596368 | Wallberg *et al.,* (2014) |
| Apis mellifera syriaca | JOR16B | SAMN02596327 | Wallberg *et al.,* (2014) |
| Apis mellifera syriaca | JOR19 | SAMN02596326 | Wallberg *et al.,* (2014) |
| Apis mellifera syriaca | JOR18 | SAMN02596325 | Wallberg *et al.,* (2014) |
| Apis mellifera syriaca | JOR17 | SAMN02596324 | Wallberg *et al.,* (2014) |
| Apis mellifera syriaca | JOR16 | SAMN02596323 | Wallberg *et al.,* (2014) |
| Apis mellifera syriaca | JOR15 | SAMN02596322 | Wallberg *et al.,* (2014) |
| Apis mellifera syriaca | JOR14 | SAMN02596321 | Wallberg *et al.,* (2014) |
| Apis mellifera syriaca | JOR13 | SAMN02596320 | Wallberg *et al.,* (2014) |
| Apis mellifera syriaca | JOR12 | SAMN02596319 | Wallberg *et al.,* (2014) |
| Apis mellifera syriaca | JOR11 | SAMN02596318 | Wallberg *et al.,* (2014) |

Table S2: Genetic diversity measures (mean ± SE) for admixed and reference populations.

| Population | Pairwise Estimator ($\hat{}$_π_) | Pairwise Estimator ($\hat{}$_π_) using called SNPs only (minor allele frequency > 0.05) | Watterson’s Estimator ($\hat{}$_w_) |
| --- | --- | --- | --- |
| African (A) | 0.0100 ± 0.0018 | 0.0047 ± 9.205e-05 | 0.0152 ± 0.0012 |
| Western European (M) | 0.0047 ± 0.0007 | 0.0028 ± 3.503e-05 | 0.0049 ± 0.0006 |
| Eastern European (C) | 0.0036 ± 0.0003 | 0.0023 ± 2.343e-05 | 0.0042 ± 0.0004 |
| Wallberg (A) | 0.0078 ± 0.0007 | 0.0039 ± 0.0001 | 0.0102 ± 0.0006 |
| Wallberg (M) | 0.0040 ± 0.0004 | 0.0027 ± 3.431e-05 | 0.0040 ± 0.0004 |
| Wallberg (C) | 0.0032 ± 0.0002 | 0.0024 ± 4.284e-05 | 0.0033 ± 0.0002 |
| Wallberg (O) | 0.0059 ± 0.0005 | 0.0037 ± 5.569e-05 | 0.0062 ± 0.0004 |
| Harpur (A) | 0.0104 ± 0.0020 | 0.0047 ± 8.898e-05 | 0.0137 ± 0.0021 |
| Harpur (M) | 0.0048 ± 0.0007 | 0.0028 ± 3.174e-05 | 0.0045 ± 0.0005 |
| Harpur (C) | 0.0035 ± 0.0003 | 0.0021 ± 1.763e-05 | 0.0041 ± 0.0004 |
| San Diego, CA, U.S.A. | 0.0096 ± 0.0018 | 0.0061 ± 8.863e-05 | 0.0108 ± 0.0020 |
| Chiapas, Mexico | 0.0109 ± 0.0023 | 0.0063 ± 0.0001 | 0.0124 ± 0.0023 |
| Guanacaste, Costa Rica | 0.0105 ± 0.0024 | 0.0061± 0.0001 | 0.0110 ± 0.0021 |
| Panamá | 0.0106 ± 0.0024 | 0.0060 ± 0.0001 | 0.0114 ± 0.0020 |
